# Эколого-генетическая оценка последствий влияния радиации на загрязненных территориях

**DOI:** 10.18699/VJ20.675

**Published:** 2020-11

**Authors:** A.B. Bigaliev, K.Z. Shalabaeva, B.E. Shimshikov, S.S. Kobegenova, L.M. Adilova, A.N. Kozhakhmetova, S. Sharakhmetov, M.N. Burkhanova

**Affiliations:** Al-Farabi Kazakh National University, Almaty, Kazakhstan; Al-Farabi Kazakh National University, Almaty, Kazakhstan; Al-Farabi Kazakh National University, Almaty, Kazakhstan; Al-Farabi Kazakh National University, Almaty, Kazakhstan; Asfendiyarov Kazakh National Medical University, Almaty, Kazakhstan; Al-Farabi Kazakh National University, Almaty, Kazakhstan; Al-Farabi Kazakh National University, Almaty, Kazakhstan; Scientific and Practical Center for Sanitary and Epidemiological Expertise and Monitoring, Almaty, Kazakhstan

**Keywords:** радиация, радионуклиды, радиоактивность, биосубстрат, полигон, мутация, цитогенетика, мутагены, экология

## Abstract

Объектами исследования являются районы Западно-Казахстанской области Республики Казах- стан, прилегающие к полигону Капустин Яр: Бокейординский, Жангалинский, Жанибекский, Казталовский, Акжаикский и Сырымский. Цель работы – радиоэкологическое обследование загрязненных территорий и ис- следование содержания загрязнителей в объектах среды, биоте (грызунах, рыбах и биосубстратах домашних животных) физико-химическими методами для оценки воздействия полигона на биоту и человека. Представ- лены результаты экспедиционных и лабораторных исследований объектов окружающей среды прилегающих к полигону районов. Приведены показатели гамма-съемки уровней радиационного фона обследуемых тер- риторий, данные о загрязнении почвы, поверхностных и подземных вод, доминантных форм растений, био- субстратов (шерсти домашних животных: верблюда, лошади, коровы). Использованы стандартные способы отбора проб, общепринятые методы исследования: радиологический и цитогенетический (микроядерный), атомно-абсорбционная спектрофотометрия. Проведено рекогносцировочное и радиоэкологическое обсле- дование объектов окружающей среды с использованием аналитических методик, что позволило определить количественное содержание токсичных компонентов, приоритетных загрязнителей и радиоактивных изо- топов. Установлено, что значения объемной активности природных и техногенных радионуклидов в пробах почвы, питьевой воды и биосубстратах (шерсти домашних животных, образцах периферической крови чело- века) из населенных пунктов соответствуют величине контрольного уровня для данного региона. Измерения гамма-излучений показали, что по периметру территории полигона и в близлежащих населенных пунктах уровень радиации находится в пределах 0.06–0.14 мкЗв/ч. Незначительное превышение уровня радиоактив- ности сохраняется вблизи падения ракет в Бокейординском районе. Исследованные районы характеризуются незначительным уровнем радиационного фона: среднее значение МЭД составляет 0.014 мкЗв/ч. Абсолютный максимум, 0.73 мкЗв/ч, зарегистрирован в пунктах падения ракет в Казталовском районе.

## Abstract (en)

The objects of study are the territory of the districts of the West Kazakhstan region of the Republic of Kazakhstan adjacent to the Kapustin Yar test site: Bokeyordinsky, Zhangalinsky, Zhanibeksky, Kaztalovsky, Akzhayiksky and Syrymsky. The purpose of the work is to conduct a radioecological survey of contaminated areas and to study the content of pollutants in environmental objects, biota (rodents, fish and biosubstrates of domestic animals) by physicochemical methods to assess the risk of the landfill’s impact on biota and humans. This paper presents the results of expeditionary and laboratory research on the pollution of environmental objects in the areas adjacent to the landfill. Indicators of the gamma-survey of the levels of radiation background of the surveyed territories as well as data on pollution of soil, surface and ground waters, dominant forms of plants, biosubstrates (hair of domestic animals: camel, horse, cow) are given. The generally accepted research methods were used: standard sampling methods, radiological, atomic adsorption spectrophotometry, cytogenetic (micronucleus) method. A reconnaissance and radioecological examination of environmental objects was carried out using analytical methods, which made it possible to determine the quantitative content of toxic components, the content of priority pollutants and radioactive isotopes. It has been established that the values of the volumetric activity of natural and man-made radionuclides in soil samples, drinking water and biosubstrates (pet hair, human peripheral blood samples) from settlements correspond to the value of the control level for this region. The measurements of radiation activity by gamma radiation showed that along the perimeter of the surveyed territory of the test site and in nearby settlements the radiation level is in the range of 0.06–0.014 μSv/h. A slight excess of the level of radioactivity persists in the area near the fall of missiles in the Bokeyordin region. The investigated regions are characterized by an insignificant level of background radiation, the average DER value for the regions as a whole is 0.14 μSv/h. The absolute maximum, 0.73 μSv/h, was recorded at the points of missile impact in the Kaztal region.

## Введение

Вокруг ракетно-ядерных полигонов в Атырауской и За- падно-Казахстанской областях Республики Казахстан сложилась сложная экологическая обстановка, вызванная радиационным загрязнением природной среды. В 2004– 2008 гг. на территории военных полигонов Капустин Яр и Азгир и в прилегающих районах проведен ряд эколо- гических работ с применением полевых и аналитических методов исследования. Тем не менее вопрос о степени влияния деятельности полигонов на окружающую среду и здоровье населения остается нерешенным^1^ (Мухаметжа- нова, 2017). На протяжении нескольких десятков лет уче- ные Казахского национального университета имени аль- Фараби проводят исследования, направленные на поиск чувствительных биологических маркеров, специфичных для оценки радиационного воздействия и информатив- ных как в раннем, так и отдаленном периоде облучения. В настоящее время одними из немногих биологических показателей (наряду с ЭПР-спектроскопией эмали зубов), в полной мере отвечающих этим требованиям, являются хромосомные аберрации в лимфоцитах периферической крови. Принципы цитогенетического метода индикации радиационного воздействия достаточно убедительно обоснованы во многих отечественных и зарубежных иссле- дованиях, результаты которых послужили основой для рекомендаций ВОЗ, МАГАТЭ и НКДАР ООН по исполь- зованию анализа хромосомных аберраций в лимфоцитах крови в качестве тест-системы для количественной оценки действия мутагенных факторов радиационной природы (WHO…, 2010). Данные о «биологической» дозе, полу- ченные с помощью цитогенетических методов шире, чем ее физическое значение, так как отражают не только результат радиационного воздействия на организм чело- века, но и его индивидуальную радиочувствительность, что позволяет более корректно прогнозировать ранние и отдаленные последствия облучения. В настоящее время в большинстве случаев, при которых люди подвергаются воздействию радиации как от естественных, так и техно- генных источников, речь идет об облучении в небольших дозах (Zhumadilov et al., 2013). Поэтому основную пробле- му составляют последствия радиационного воздействия в малых дозах, особенности биологических эффектов которого до сих пор являются предметом активных дис- куссий (Hоshi, Saimova, 2017).

^1^ Национальный доклад о состоянии окружающей среды и использовании природных ресурсов Республики Казахстан за 2011–2014 годы. Под ред. РГП на ПХВ «Информационно-аналитический центр охраны окружающей среды». Алматы, 2015;214.

Актуальность предлагаемой работы определена необходимостью изучения современного состояния природных популяций растений, животных и человека в условиях различного фона ионизирующей радиации при длитель- ном хроническом облучении в местах ядерных испытаний на территории полигона Капустин Яр. В свою очередь, оценка влияния деятельности полигона требует разработ- ки природоохранных мероприятий по уменьшению техно- генного воздействия, ранней диагностики устойчивости генома природных популяций, подвергающихся давлению антропогенного пресса. При этом требуются учет и про- гнозирование текущих и отдаленных последствий влия- ния факторов среды обитания на биоту и здоровье населения, что представляет чрезвычайную актуальность для данного региона и имеет научно-практическую значи- мость (Bigaliev, 2016; Markabayeva et al., 2018).

## Материалы и методы

Использованы общепринятые методы отбора проб почвы, воды, образцов растений, животных и человека. Отбор проб почвы производили согласно стандартной методике № 5.05.008-99 г. (Островская и др., 2014). Пробы почвы плотно запечатывали в кюветах и перед спектрометри- ческими измерениями выдерживали в течение 2–3 нед. для установления подвижного равновесия между изото- пами ^226^Ra, ^224^Ra и продуктами их распада (^222^Ra, ^220^Ra и др.). Исследования проводили согласно утвержденному и внесенному в государственный реестр Республики Казахстан документу «Методика измерения активности радионуклидов с использованием сцинтилляционного гамма-спектрометра с программным обеспечением «Про- гресс» (рег. № KZ.07.00.00304-2014)^2^. Из населенных пунктов, расположенных на прилегающей к полигону тер- ритории, отобраны биосубстраты домашних животных, образцы доминантных видов растений, взяты образцы периферической крови жителей. Все они являются звеньями трофической цепи питания и участвуют в миграции радионуклидов и тяжелых металлов (Постановление Правительства РК «Об утверждении Правил объявления чрезвычайной экологической ситуации» № 431 от 5 мая 2005 г.). Определение активности радионуклидов про- водили спектрометрическим методом с использованием гамма-спектрометров «МКС-01А Мультирад» (ООО «НТЦ «Амплитуда», Россия), Canberra CR-4018 (США). Для выявления содержания тяжелых металлов применена атомно-абсорбционная спектрофотометрия. Исследования проведены в физико-химической лаборатории факультета биологии и биотехнологии Казахского национального университета имени аль-Фараби в соответствии с обще- принятыми стандартами (ГОСТ 26929-86, ГОСТ 30178-96, ИСО 8288-1986) на спектрофотометре «МГА-915МД» (ГК «Люмэкс», Россия) (Техногенные потоки…, 2001).

^2^ Инструкция по отбору проб почвы при радиационном обследовании за- грязненной территории, 1987.

Для цитогенетического исследования использовали микроядерный тест. Цитологические препараты для микроядерного анализа готовили согласно общепринятым ме-тодикам (Руководство…, 2002), фотографировали в све- товых микроскопах Axioskop 40 (Carl Zeiss, Германия) и Micro Optic (Австрия). Забор образцов крови для микроядерного анализа проводили из фаланги пальцев в условиях сельских поликлиник, соблюдая принципы анти- септики. Объектом исследования служила популяция ко- ренных жителей, включающая 107 человек, проживающих на территории, прилегающей к полигону Капустин Яр.

## Результаты и обсуждение

Планомерные работы по оценке влияния деятельности полигонов Капустин Яр и Азгир на окружающую среду начались в 2001 г., став первым этапом программы иссле- дований в регионе, охватывающем шесть южных районов Западно-Казахстанской области и два района Атырау- ской области. Работы первого этапа завершены в 2002 г. (Loomis et al., 1990) – представлены результаты полевых и лабораторных исследований в Бокейординском и южной части Жангалинского районов. Выполнены инструмен- тальные измерения радиационных параметров территории данных районов, используемой для падения отделяющих- ся частей ракет. Однако изучение состояния здоровья на- селения проведено только с использованием медицинской и демографической статистики. Данные медицинских исследований получены разными составами врачей, без сравнения с контрольным районом и проведения необ- ходимых генетических исследований (популяционных и цитогенетических), должной статистической обработки полученных результатов.

В 2018–2019 гг. мы впервые взяли пробы, образцы и биосубстраты в этих же районах Западно-Казахстанской области для лабораторных исследований. Для рекогносци- ровочного изучения загрязненных участков, выявленных по результатам предыдущих исследований, проведено экспедиционное обследование экологического состояния компонентов окружающей среды: произведен отбор проб почвы, воды (подземных и поверхностных), растений, биосубстратов (шерсти домашних животных, органов и тканей рыб, грызунов, ящериц, образцов периферической крови жителей). В 2018 г. в Жангалинском районе взято 78 проб и образцов, Бокейординском районе – 89, Жанибекском районе – 75, Казталовском районе – 91. Всего по четырем районам – 333. В 2019 г. в Акжаикском районе отобрано 78 проб и образцов, бывшем Тайпакском районе – 89, Сырымском районе – 46. Всего по трем районам – 213. Проведены измерения радиометрических параметров среды в точках отбора проб в соответствии с методикой гамма-съемки^3^.

^3^Коллектив авторов ИРБЭ НЯЦ РК – отчет по теме: «Оценка влияния дея- тельности военных полигонов на окружающую среду и определение мер по ее реабилитации», 2002.

**Полевые исследования**

Результаты радиологического исследования активности радионуклидов техногенного происхождения в пробах и образцах тест-объектов, взятых с территорий зоны по- лигона, представлены в табл. 1.

**Table 1.**
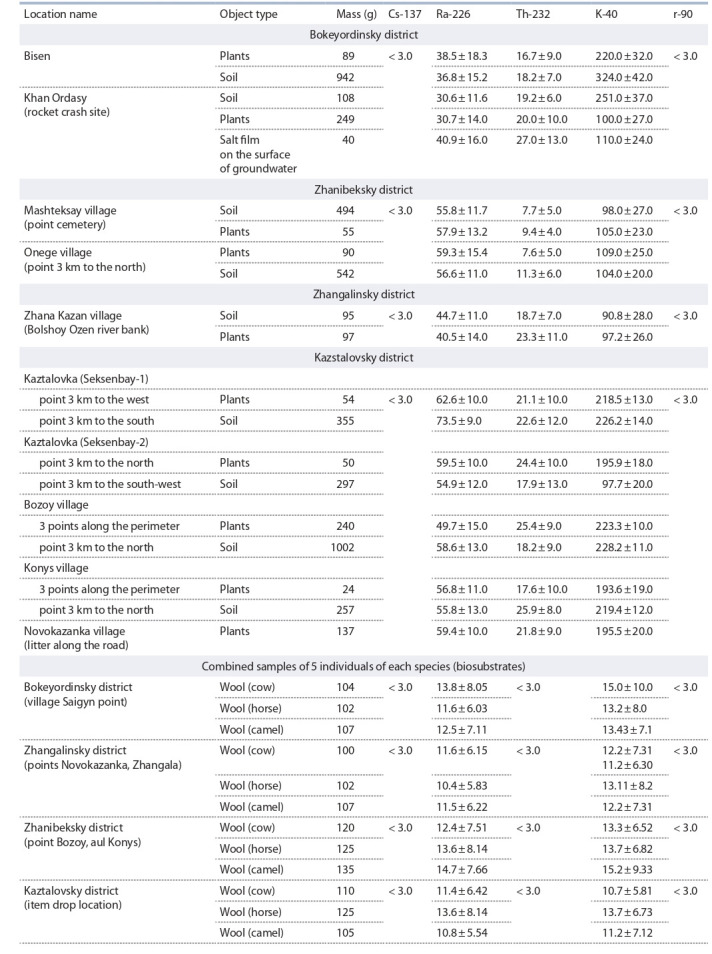
Activity of technogenic radionuclides in samples and samples of test objects

Проведенные радиоэкологические исследования объек- тов окружающей среды с использованием аналитических методик позволили определить количественное содержа- ние приоритетных загрязнителей и радиоактивных изо- топов. Установлено, что значения объемной активности природных и техногенных радионуклидов в пробах почвы, питьевой воды и биосубстратах (шерсти домашних живот- ных) из исследуемых населенных пунктов соответствуют показателям контрольного уровня. Измерения радиаци- онной активности методом гамма-излучения показали, что по периметру обследованной территории полигона и в близлежащих населенных пунктах уровень радиации находится в пределах 0.06–0.014 мкЗв/ч, что соответствует уровню радиации для данного региона. Незначительное превышение уровня радиоактивности сохраняется на тер- ритории вблизи падения ракет в Бокейординском районе. Таким образом, исследованные районы характеризуются незначительным уровнем радиационного фона, среднее значение МЭД составляет 0.14 мкЗв/ч (Постановление Правительства РК «Об утверждении Правил объявления чрезвычайной экологической ситуации» № 431 от 5 мая 2005 г.). Абсолютный максимум, 0.73 мкЗв/ч, зарегистри- рован в пунктах падения ракет в Казталовском районе.

Определено содержание тяжелых металлов в подготовленных пробах (почвы, растений и воды) методом атомно-абсорбционной спектрометрии согласно ГОСТ СТ РК ИСО 11047-2008 на приборах «МГА-915» и АSS-1 (табл. 2).

**Table 2.**
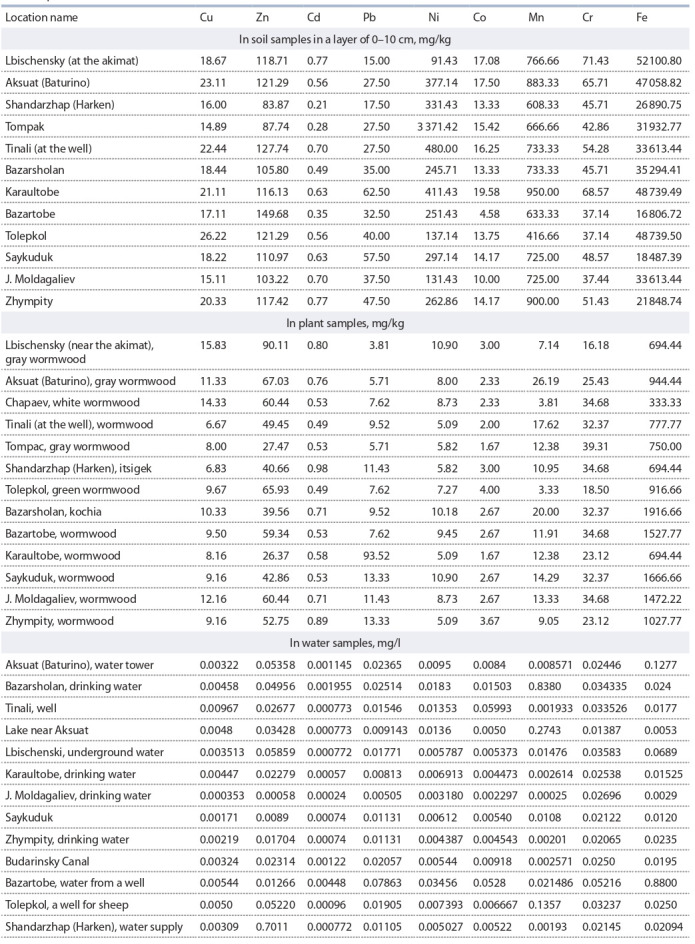
The content of heavy metals in soil, plant and water (surface and underground) samples in the Kapustin Yar landf ill area

Наибольшая концентрация тяжелых металлов выявлена в подземных водах обследованных районов. Так, максимальные показатели содержания Zn составили 0.05– 0.71 мг/л, Mn – 0.838 (с. Базаршолан), Cr – 0.052, Fe – 0.06–0.88 мг/л (с. Базартобе).

Незначительно увеличенное содержание тяжелых металлов определено на территориях с относительно повы- шенным радиационным фоном (с. Харкен, Базаршолан, Базартобе) – как в образцах почвы, так и растений. Зна- чительное накопление тяжелых металлов Ni, Zn, Co, Cr установлено в почвах обследованных территорий, прилегающих к полигону. Полученные результаты позволяют констатировать, что загрязнение окружающей среды пред- ставляет определенную опасность для биоты и человека. К стойким химическим загрязнителям кумулятивного действия со специфическими токсическими свойствами относятся прежде всего тяжелые металлы. Тройку наибо- лее экологически опасных тяжелых металлов составляют свинец, ртуть и кадмий. Данные металлы являются потен- циально опасными токсикантами, способными вызывать нарушения жизнедеятельности водной и наземной биоты, и, следовательно, могут быть дестабилизирующим фак- тором в экологической системе сложившегося биоценоза (Фомин Г.С., Фомин А.Г., 2001). Также общеизвестно, что тяжелые металлы, накапливаясь в тканях организма, из- меняют транскрипционную активность хромосом (Теплая, 2013; Chaizhunusova et al., 2017; Serzhanova et al., 2018) и приводят к нарушениям устойчивости генома. Обна- руженный относительно высокий уровень активности радионуклидов в локальных точках среди исследуемых тест-объектов отмечается в почвах, подземных водах, рас- тениях и биосубстратах в пределах контрольных величин.

**Цитогенетические исследования**

В популяциях человека и животных имеются индивиду- умы и особи с различной устойчивостью к мутагенным факторам. Особое внимание в этой связи следует обратить на виды с выраженной нестабильностью генома (Bigalyev et al., 2014).

**Микроядерный анализ образцов рыб.** Из образцов отловленных на исследуемой территории видов рыб были приготовлены гематологические препараты для микро- ядерного теста. У обследованных рыб эритроциты были представлены молодыми бластными формами и зрелыми клетками (см. рисунок). Молодые клетки в зависимости от степени развития представляли собой круглые или слегка вытянутые клетки, размеры ядер которых варьировали от крупных, занимавших большую часть клетки, до мелких. В большинстве случаев зрелые эритроциты имели эллип- совидную форму, вытянутое ядро красно-фиолетового цвета, прозрачную цитоплазму серо-розового цвета. На- ряду со здоровыми клетками были зарегистрированы и патологические.

**Fig. 1.**
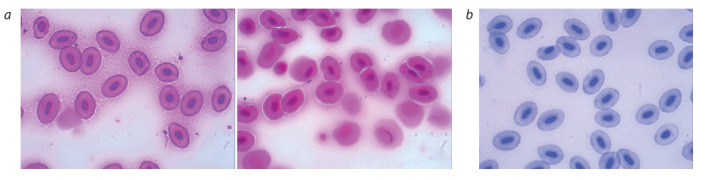
Pathologies of erythrocytes of crucian carp: a – micronuclei, displacement of the nucleus to the periphery, anisacytosis; b – micronuclei, invagination of the nucleus (× 1000, coloration with basic fuchsin according to Pfeffer).

**Вобла.** Клетки крови воблы в большинстве случаев характеризовались неправильной формой эритроцитов. Патологии эритроцитов включали микроядра, смещение ядра к периферии, анизоцитоз, инвагинацию ядра, что несколько затрудняло проведение микроядерного анализа.

**Судак.** У особей судака умеренно часто встречались патологии двух групп: смещение ядра к периферии, вы- зываемое набуханием клетки, микроядра, возникающие при нарушениях клеточного деления, и инвагинация ядра, являющаяся маркером дегенерации эритроцитов. Отме- чены единичные очаги ядерных теней, возникающих при разрушении эритроцитов.

**Жерех.** У особей жереха были наиболее выражены патологии первой группы. Отмечено большое количество эритроцитов неправильной формы – грушевидной, сер- повидной, пятиугольной (пойкилоцитоз). Незначительно представлены ядерные патологии в виде инвагинации ядра и микроядер.

**Карась.** В эритроцитах изученных карасей присутство- вали как относительно крупные, так и мелкие микроядра. При этом в одной клетке в некоторых случаях наблюда- лось по одной-две и более микроядер. Однако наиболее часто встречались клетки с одним микроядром помимо основного ядра. Кроме того, наблюдались смещение ядра к периферии клетке и инвагинация ядра (см. рисунок).

Нарушения эритроцитов, выявленные у исследуемых видов, свидетельствовали о дестабилизации физиологических процессов в организме обследованных рыб, приводящей к развитию патологии митоза. Так, анизо- и пойкилоцитоз показывают функциональную недостаточность кроветворных органов, а также наблюдаются при анемии. Наряду с вышеуказанными патологиями обнаружено сме- щение ядер к периферии (возникающее при набухании). К дегенеративным изменениям также можно отнести инвагинацию ядра, свидетельствующую о деградации самого эритроцита. Последующий микроядерный анализ мазков показал увеличение спонтанной частоты клеток с микроядрами в 1.5–2 раза в загрязненных районах. Вероятность встречаемости эритроцитов с микроядрами в периферической крови при спонтанном мутагенезе со- ставляет 0.5–1.0 % (Fenech, 2011).

Полученные результаты в дальнейшем будут сопо- ставлены с результатами гистопатологического анализа внутренних органов (жабр, печени, кишечника, мышц и гонад) для оценки токсикологического состояния среды обитания рыб.

**Микроядерный анализ клеток человека.** Объектом исследования служила свежеполученная кровь из пальца смешанной популяции, включающей 107 человек, прожи- вающих в районах с неблагополучной экологической об- становкой из-за близости полигона Капустин Яр. Сводные группы для микроядерного анализа составили 23 человека с наследственными дегенеративными заболеваниями нервной системы, 27 – с гепатоцеребральной дистрофией, 24 – с врожденными пороками развития, 25 – с перина- тальной патологией, 8 – с синдромом Дауна. Средний возраст больных – 33.9 года. В контрольную группу вошли 50 практически здоровых людей в возрасте 20–37 лет. Результаты микроядерного теста представлены в табл. 3.

**Table 3.**
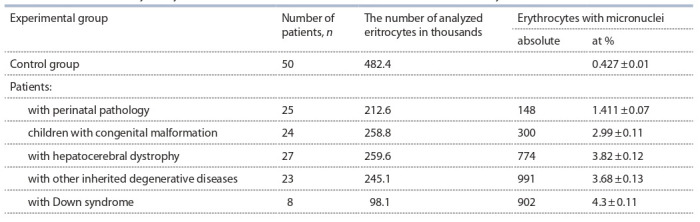
The number of erythrocytes with micronuclei in the blood of individuals from the study area

Количество выявленных микроядер достоверно выше у пациентов с наследственно-дегенеративными заболевани- ями (t = 21.68, р < 0.01), гепатоцеребральной дистрофией (t = 33.93, р < 0.01), врожденными пороками развития (t = 25.63, р < 0.01), синдромом Дауна (t = 38.73, p < 0.01) и перинатальной патологией (t = 14.05, p < 0.01) в сравнении с контрольной группой – 0.427 ± 0.01.

По данным различных авторов, спонтанный уровень микроядер у здоровых людей колеблется от 0.24 ± 0.01 до 0.34 ± 0.1 %. Больные с высоким показателем микроядер обследованы повторно через месяц, у них обнаружена тенденция повышения количества микроядер в эритро- цитах периферической крови.

В зависимости от размера микроядра распределены на две группы: крупные и мелкие. Эритроциты с мелкими микроядрами составляли 88 %, а с крупными микроядра- ми – 12 %. Типы нарушений ядер соматических клеток различаются по количеству и форме в зависимости от видовой, тканевой принадлежности. По данным авторов, высокий уровень эритроцитов с микроядрами зареги- стрирован у больных с различными формами миопатии. Дальнейшие исследования позволили прийти к выводу, что нестабильность генома подтверждается и другими тестами (Ильинских и др., 1992). Проведен корреляци- онный анализ, который свидетельствует, что образование крупных микроядер тесно связано с геномными наруше- ниями хромосомного аппарата (r = 0.70, р < 0.05), тогда как уровень клеток с мелкими микроядрами коррелирует с частотой нарушений в структуре хромосом (r = 0.60, р < 0.05). Установлено, что частота эритроцитов с мелки- ми микроядрами не зависит от уровня патологии митоза, многогрупповых мета- и анафаз с мостами (во всех слу- чаях р > 0.05). Уровень клеток с крупными микроядрами тесно связан с патологией митоза – отставанием отдель- ных хромосом в мета- и анафазах – и свидетельствует о том, что крупные микроядра, по-видимому, образованы отставшими хромосомами, в то время как мелкие – в основном структурными аберрациями хромосом. Авто- ры утверждают, что приведенные данные показывают тесную связь между цитогенетическими нарушениями и образованием микроядер (Djokovic-Davidovic et al., 2016).

## Заключение

Значения объемной активности природных и техноген- ных радионуклидов в пробах почвы, питьевой воды, биосубстратах (шерсти домашних животных и образцах периферической крови человека) из населенных пунктов, расположенных рядом с полигоном Капустин Яр, соот- ветствуют величине контрольного для Западно-Казах- станской области уровня (радиационный фон в пределах 0.06–0.014 мкЗв/ч). Незначительное превышение уровня радиоактивности сохраняется на территории вблизи па- дения ракет в Бокейординском районе. Цитогенетические исследования с использованием микроядерного теста в со- матических клетках рыб показали увеличение спонтанной частоты клеток с микроядрами в 1.5–2 раза в загрязнен- ных районах. Вероятность встречаемости эритроцитов с микроядрами в периферической крови при спонтанном мутагенезе составляет 0.5–1.0 %. Анализ мазков крови человека на микроядерный тест также показал увеличение спонтанной частоты клеток с микроядрами в 1.5–2 раза в загрязненных районах по сравнению со спонтанной частотой 0.5–1.0 %.

## Conflict of interest

The authors declare no conflict of interest.
